# Toward a large‐scale and deep phenological stage annotation of herbarium specimens: Case studies from temperate, tropical, and equatorial floras

**DOI:** 10.1002/aps3.1233

**Published:** 2019-03-20

**Authors:** Titouan Lorieul, Katelin D. Pearson, Elizabeth R. Ellwood, Hervé Goëau, Jean‐Francois Molino, Patrick W. Sweeney, Jennifer M. Yost, Joel Sachs, Erick Mata‐Montero, Gil Nelson, Pamela S. Soltis, Pierre Bonnet, Alexis Joly

**Affiliations:** ^1^ University of Montpellier Montpellier CEDEX 5 France; ^2^ Institut national de recherche en informatique et en automatique (INRIA) Sophia‐Antipolis, ZENITH team, Laboratory of Informatics Robotics and Microelectronics–Joint Research Unit, 34095 Montpellier CEDEX 5 France; ^3^ Department of Biological Science Florida State University 319 Stadium Drive Tallahassee Florida 32306 USA; ^4^ La Brea Tar Pits and Museum Natural History Museum of Los Angeles County 5801 Wilshire Boulevard Los Angeles California 90036 USA; ^5^ AMAP Université de Montpellier CIRAD, CNRS, INRA, IRD Montpellier France; ^6^ CIRAD, UMR AMAP Montpellier France; ^7^ Division of Botany Peabody Museum of Natural History Yale University P.O. Box 208118 New Haven Connecticut 06520 USA; ^8^ Department of Biological Sciences California Polytechnic State University 1 Grand Avenue San Luis Obispo California 93407 USA; ^9^ Agriculture and Agri‐Food Canada Ottawa Canada; ^10^ School of Computing Costa Rica Institute of Technology Cartago Costa Rica; ^11^ iDigBio Florida State University Tallahassee Florida 32306 USA; ^12^ Florida Museum of Natural History University of Florida Gainesville Florida 32611 USA

**Keywords:** convolutional neural network, deep learning, herbarium data, natural history collections, phenological stage annotation, visual data classification

## Abstract

**Premise of the Study:**

Phenological annotation models computed on large‐scale herbarium data sets were developed and tested in this study.

**Methods:**

Herbarium specimens represent a significant resource with which to study plant phenology. Nevertheless, phenological annotation of herbarium specimens is time‐consuming, requires substantial human investment, and is difficult to mobilize at large taxonomic scales. We created and evaluated new methods based on deep learning techniques to automate annotation of phenological stages and tested these methods on four herbarium data sets representing temperate, tropical, and equatorial American floras.

**Results:**

Deep learning allowed correct detection of fertile material with an accuracy of 96.3%. Accuracy was slightly decreased for finer‐scale information (84.3% for flower and 80.5% for fruit detection).

**Discussion:**

The method described has the potential to allow fine‐grained phenological annotation of herbarium specimens at large ecological scales. Deeper investigation regarding the taxonomic scalability of this approach is needed.

Global changes that threaten biodiversity are numerous and rapidly increasing. To reduce the human impact on biodiversity loss, the scientific community must develop new, multi‐disciplinary approaches that incorporate the most recent advances in biodiversity informatics, large occurrence and trait data sets, and large‐scale taxonomic and ecological analyses. The development of the Open Science movement, citizen science initiatives, global digitization of natural history collections, and online cyberinfrastructure provide new opportunities for mobilizing and integrating massive amounts of biological data, driving the discovery of complex patterns and new hypotheses for further study (Soltis and Soltis, 2016; Allen et al., 2018). This large‐scale data integration across disciplines, continents, and infrastructures allows new investigations in ecology, systematics, and evolution that offer the capacity to make biodiversity projections and provide crucial information for scientists and other stakeholders (e.g., land use managers, policy makers, agricultural producers, mining contractors). In this study, we investigate means to enable such data integration within the context of phenological studies using digitized herbarium specimens.

Herbarium specimens are dried and pressed plants or parts of plants that have been mounted on archival paper; labeled with data about, e.g., the identification of the plant, collection locality, collection date, and collector; and stored in natural history collections called herbaria. These plant specimens provide crucial data for the study of plant diversity, ecology, evolution, genetics, and biodiversity, to name only a few (Graham et al., 2004). When herbarium specimens are “digitized”—converted into a digital format by imaging and transcription of label data—they have even greater potential for answering major research questions related to the recent impact of humanity on biodiversity (Davis et al., 2015; Soltis, 2017; James et al., 2018; Meineke et al., 2018; Soltis et al., 2018). These millions of herbarium records have accumulated a valuable heritage and knowledge of plants over centuries, across all continents. Recent ambitious initiatives in the United States, Australia, Brazil, and Europe are digitizing this information and making it available online to the scientific community and general public. Herbarium‐based phenological research offers the potential to provide novel insights into plant diversity and ecosystem processes under future climate change (Zalamea et al., 2011; Willis et al., 2017; Yost et al., 2018).

Rapid human‐induced climate change has affected plant phenology over the past century, with likely impacts on reproductive success, plant–pollinator interactions, and even carbon and nutrient cycling (Menzel et al., 2006; Gordo and Sanz, 2010; Bartomeus et al., 2011; Ellwood et al., 2013; Primack et al., 2015). However, the study of phenological shifts is only possible with historical and long‐term data sets that can establish the phenological patterns of plants before human‐induced climate change. Herbarium data sets are therefore essential as unique, verifiable sources of historic information on species localities and phenological states. Most phenological studies are based on individual and manual phenological evaluation conducted by researchers or a small number of professionals, which is a laborious and resource‐intensive process.

Annotating the tens of millions of existing digitized specimens for phenology requires an unrealistic amount of work for professional botanists to carry out in a reasonable time. Citizen scientists are capable of making substantial contributions to digital biodiversity data (Ellwood et al., 2018); however, using citizen science data for ecological studies often requires complementary annotations to ensure data quality. A remarkable example of the complementary contributions provided by automated and volunteer classifications is provided by Jones et al. (2018) who have used them jointly to automatically identify wild animals from camera traps.

Automated approaches, such as computer vision and machine learning methods, can complement valuable citizen science data and may help bridge the “annotation gap” (Unger et al., 2016) between existing data and research‐ready data sets. Deep learning approaches, in particular, have been recently shown to achieve impressive performance on a variety of predictive tasks such as species identification (Joly et al., 2017; Wäldchen et al., 2018), plant trait recognition (Younis et al., 2018), plant species distribution modeling (Botella et al., 2018), and weed detection (Milioto et al., 2018). Carranza‐Rojas et al. (2017, 2018a) reported the first attempts to use deep learning to tackle the difficult task of identifying species in large natural history collections and showed that convolutional neural networks trained on thousands of digitized herbarium sheets are able to learn highly discriminative patterns from pressed and dried specimens. These results are very promising for extracting a broad range of other expert annotations in a fully automated way. However, as with any statistical learning method, convolutional neural networks are sensitive to bias issues, including the way in which the training data sets are built (Carranza‐Rojas et al., 2018b), necessitating methodological considerations to avoid bias and misleading conclusions. Moreover, as good as the prediction might be on average, the quality of the produced annotations can be very heterogeneous from one sample to another, depending on various factors such as, e.g., the morphology of the species, the storage conditions in which the specimen was preserved, the age of the specimen, or the skill of the annotator.

The goal of this study is to evaluate the capacity of deep learning technologies for large‐scale phenological annotation of herbarium data. We test new methods and algorithms to automate the scoring of reproductive phenological stages within a huge amount of digitized material, to provide significant resources for the ecological and organismal scientific communities. Specifically, we aim to answer three questions: (1) Can fertility, i.e., presence of reproductive structures, be automatically detected from digitized specimens using deep learning? (2) Are the detection models generalizable to different herbarium data sets? and (3) Is it possible to finely automatically record stages (i.e., phenophases) within longer phenological events on herbarium specimens? To our knowledge, this is the first time that such an analysis has been conducted at this scale, on such a large number of herbarium specimens and species. A study at this taxonomic scale reveals the opportunities and limits of such an approach for large ecological studies, which are discussed in the following sections.

## METHODS

### Data sets

To evaluate our approach at different levels (in terms of information precision) and on different floras (from temperate to equatorial), four data sets of specimens from American herbaria were used in this study.

Three data sets consist of selected specimens from herbaria located in different geographic and environmental regions. Each specimen of these three data sets was annotated with the following fields: family, genus, species name, fertile/non‐fertile, presence/absence of flower(s), and presence/absence of fruit(s). Based on a data curation pipeline, our resulting data set was composed of 163,233 herbarium specimens belonging to 7782 species, 1906 genera, and 236 families. Specimens were annotated as “fertile” if any reproductive structures were present, such as sporangia (ferns), cones (gymnosperms), flowers, or fruits (angiosperms). Non‐fertile specimens were those that lacked any reproductive structures. Most herbarium specimens in this study were annotated by herbarium assistants, curators, technicians, or other personnel responsible for digitizing specimen label data (e.g., trained undergraduate student workers), often long after the collection event. Collectors may have included the phenological status of the sampled plant or population on the specimen label or in the field notes used to create the label, in which case the digitization technician may have annotated the specimen record accordingly. More often than not, however, the digitization technician must determine from the specimen whether reproductive structures are present. Occasionally, specimens are annotated after digitization, e.g., for specific research projects, in subsequent data quality steps, or with further identifications of the specimen. A detailed description of the herbarium specimen annotation process is provided in Appendix 1.

The fourth data set consists of 20,371 herbarium specimens from 11 genera in the sunflower family (Asteraceae). These specimens were annotated by one co‐author (K.D.P.) for a study of phenological trends in the southeastern United States (Pearson, 2019a). The distinction of this data set from the other three data sets is that (1) it is annotated with fine‐grained phenophase scores rather than presence/absence attributes (see description below), (2) it is annotated by one person only and not a diversity of persons distributed in different herbaria, and (3) all specimens were annotated from images of digitized specimens rather than from physical herbarium specimens or the wild plant.

Each of these data sets is described below and presented in Table 1:

**Table 1:** Description of data sets used on EXP1‐Fertility, EXP2‐Fl.Fr, and EXP3‐Pheno.^a^

Full data set names	Data set acronyms	No. of herbarium specimens	Fertile proportion	Flower proportion	Fruit proportion	No. of families	No. of genera	No. of species
New England Vascular Plant specimens	NEVP	42,658	90.9%	64.9%	34.9%	16	340	1375
Florida State University's Robert K. Godfrey Herbarium	FSU	54,263	92.7%	73.9%	55.2%	202	1189	3870
IRD Herbarium of Cayenne	CAY	66,312	79.4%	46.6%	35.1%	126	764	3024
Asteraceae phenophase data set	PHENO	20,994	100%	NA	NA	1	16	139

Note

IRD = Institut de Recherche pour le Développement; NA = not available.

a See Appendices 2 and 3 for the lists of institutions contributing data to the NEVP and PHENO data sets.

**Figure 1:**
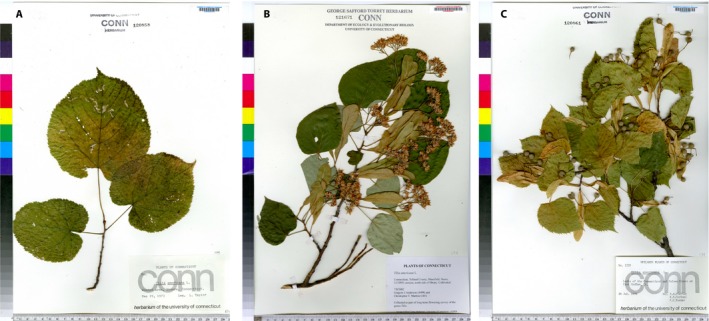
Illustration of the different phenological stages of *Tilia americana* on the NEVP herbarium data set. (A) Non‐fertile specimen, (B) specimen with open flowers, (C) specimen with ripe fruits.

**Figure 2:**
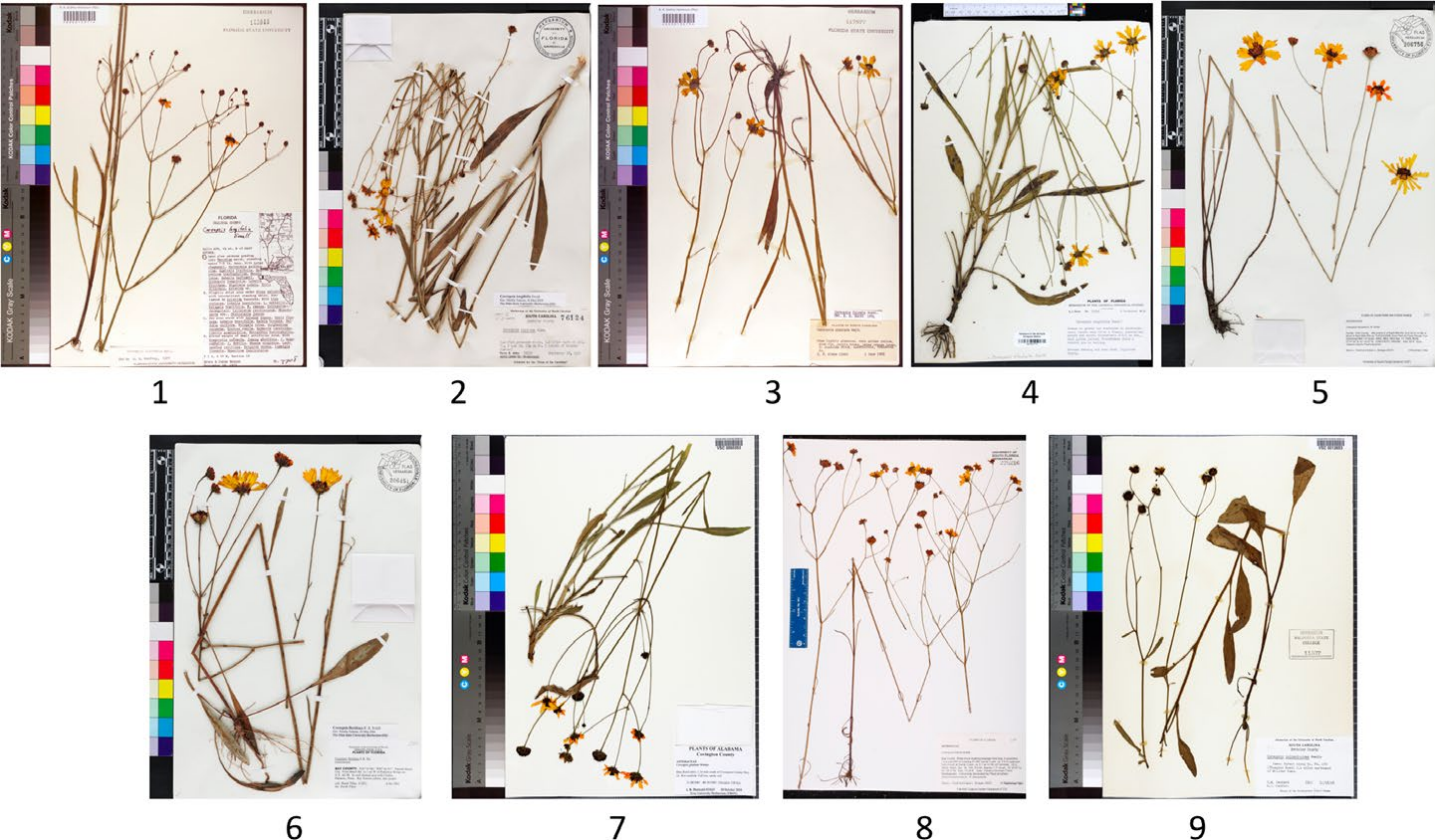
Illustration of the nine different phenophases of *Coreopsis gladiata* recorded in the PHENO data set.

- NEVP: This data set of specimens from the New England Vascular Plant (NEVP) project was produced by members of the Consortium of Northeastern Herbaria (http://neherbaria.org/). The data set comprises 42,658 digitized specimens that belong to 1375 species and come from several North American institutions (listed in Appendix 2). Most of the specimens in this data set are from the north‐temperate region of the northeastern United States. Figure 1 provides an illustration of the different phenological stages recorded for *Tilia americana* L. in this data set.
- FSU: This data set was produced by the Florida State University's Robert K. Godfrey Herbarium (FSU; http://herbarium.bio.fsu.edu/), a collection that focuses on northern Florida and the U.S. southeast coastal plain, one of North America's biodiversity hotspots (Noss et al., 2015). This data set contains 54,263 digitized herbarium specimen records that belong to 3870 species, making it the taxonomically richest data set in this study. Most species in this data set grow under subtropical or warm temperate conditions in the southeastern United States.
- CAY: This data set comes from the Institut de Recherche pour le Développement's (IRD) Herbarium of French Guiana (CAY; http://herbier-guyane.ird.fr/). CAY is dedicated to the Guayana Shield flora, with a strong focus on tropical tree species. This data set is composed of 66,312 herbarium specimens that belong to 3024 species. All digitized specimens of this herbarium are accessible online (http://publish.plantnet-project.org/project/caypub). Most specimens were collected in the tropical rainforests of French Guiana, with the remaining specimens coming mostly from Suriname and Guyana.
- PHENO: This data set includes 20,371 herbarium specimens of 139 species in the Asteraceae produced in a study of phenological trends in the U.S. southeast coastal plain. The data set is composed of specimen records from 57 herbaria (Appendix 3). Each recorded specimen was annotated by K.D.P. for quartile percentages (0%, 25%, 50%, 75%, or 100%) of (1) closed buds, (2) buds transformed into flowers, and (3) fruits. According to the distribution of these three categories for each specimen, a phenophase code was computed. The method used to compute this code is provided in Table 2. Figure 2 provides an illustration of the nine phenophases recorded for *Coreopsis gladiata* Walter in this data set.

**Table 2:** Phenophases assigned to specimens in the PHENO data set with percentages of reproductive structures on a specimen that are closed buds, flowers, and fruits

Phenophase code	Phenophase description	Distribution^a^
1	Specimen with unopened flowers	100% closed buds
2	Specimen mainly in buds	75% closed buds, 25% flowers, 0% fruits 75% closed buds, 0% flowers, 25% fruits
3	Specimen essentially in buds and flowers	50% closed buds, 50% flowers, 0% fruits
4	Specimen mainly in buds and flowers	50% closed buds, 25% flowers, 25% fruits 25% closed buds, 75% flowers, 0% fruits
5	Specimen mainly in flowers	0% closed buds, 100% flowers, 0% fruits 25% closed buds, 50% flowers, 25% fruits
6	Specimen mainly in flowers and fruits	0% closed buds, 75% flowers, 25% fruits25% closed buds, 25% flowers, 50% fruits50% closed buds, 0% flowers, 50% fruits
7	Specimen essentially in flowers and fruits	0% closed buds, 50% flowers, 50% fruits
8	Specimen mainly in fruits	0% closed buds, 25% flowers, 75% fruits25% closed buds, 0% flowers, 75% fruits
9	Specimen essentially in fruits	0% closed buds, 0% flowers, 100% fruits

a Distribution of closed buds, flowers, and fruit on the specimen.

### Evaluated deep learning framework

We considered each of our experiments as a classification task, and we focused on the use of convolutional neural networks (CNNs), which have been shown to considerably improve the accuracy of automated visual classification of botanical data compared to previous methods (Wäldchen and Mäder, 2018). The main strength of this technology is the ability to learn discriminant visual features directly from the raw pixels of the images without being negatively impacted by the high dimensionality of the input data. Because the sizes of our data sets are relatively small for training these types of models (tens of thousands and hundreds of thousands compared to tens of millions usually required), we used transfer learning techniques (Shin et al., 2016) to improve our models.

We took a ResNet50 network (He et al., 2016) pre‐trained on tens of millions of images from the ImageNet data set (Deng et al., 2009) and fine‐tuned it on our data sets. ResNet50 was chosen because it is a state‐of‐the‐art model, and it is widely used in image classification tasks. Moreover, pre‐trained parameters can be easily found for most Deep Learning frameworks, in particular, for PyTorch (https://pytorch.org/), which we used for the experiments. Although the input image size of the original ResNet50 model was fixed to 224 × 224 pixels, herbarium specimens are usually digitized at high resolution to record fine visual information. To cope with the rectangular shape of these specimens and to preserve as much detail as possible, we adapted the ResNet50 model to use higher‐resolution images. This is possible because the convolutional layers of the model are not constrained by the input image size; we can thus retain them while only modifying the final pooling layer to operate on all spatial dimensions and retraining the final classification layer from scratch. Although the dimension of the images is increased, this modification does not change the number of parameters in the network. Details on this model adaptation are provided in Appendix 4.

We tested two resolutions: 400 × 250 pixels and 850 × 550 pixels, which are both significantly larger than the usual resolution of CNNs (in most cases less than 300 × 300 pixels). In the following sections, we denote these resolutions as, respectively, ResNet50‐Large and ResNet50‐VeryLarge. To increase the volume and visual diversity of the training data set, we also performed data augmentation by performing random horizontal and vertical flips and random rotations of ±45 degrees on the input images. Data augmentation is a well‐known strategy to improve the invariance of neural networks to acquisition conditions and improve their performances. To choose the hyperparameters, we retained 10% of the training set for validation purposes only. A complete description of the fine‐tuning procedure with all hyperparameters is provided in Appendix 5.

### Description of experiments

#### Assessing performance of deep learning models

Three experiments were conducted in this study to evaluate the performances of four different models for automated annotation of phenology on herbarium specimens. The first two experiments used the three herbarium data sets (NEVP, FSU, CAY), and the last used the PHENO data set.

1. The first experiment (EXP1‐Fertility) aimed to evaluate the capacity of the CNN to detect fertile material (i.e., specimens with reproductive structures present), based on the analysis of three test sets (A, B, C). Test set A (Random‐split) was a random set of herbarium specimens that were not used as training data of the CNN model but that belonged to species and collections represented in the training data. This test set of 13,415 specimens allowed us to evaluate a scenario in which one herbarium collection uses its annotated specimens to train a model that automatically annotates the un‐annotated specimens. Test set B (Species‐split) was a selection of herbarium specimens belonging to species that were not present in the training data. For species selection, we first ordered species by decreasing number of herbarium specimens. We then selected from the full species list, one species out of 10, starting at the tenth. All specimens belonging to the species selected were used as test data. This test set of 14,539 specimens allowed us to evaluate a scenario in which the trained model must annotate specimens belonging to species that were never used for the training phase. This is important to evaluate as herbarium collections regularly receive specimens of species that were not previously in their collections. Test set C (Herbarium‐split) was a selection of herbarium specimens from nine herbaria (Boston University, Central Connecticut State University, Keene State College, New York Botanical Garden, University of Maine, University of Massachusetts, University of Rhode Island, University of Vermont, and Western Connecticut State University) of the NEVP data set that were not present in the training data. For herbaria selection, we first ordered them by decreasing number of herbarium specimens. We then selected one herbarium out of every two from the full herbaria list, starting at the second. All specimens belonging to the herbaria selected were used as test data. This test set of 14,540 specimens enabled evaluation of trained model performance for entirely new herbarium collections. Each collection has its own methodology for mounting plants (e.g., with particular glue or thread), as well as unique labels, annotations, imaging scale bars, and stamps, that can potentially influence annotation performance. Test set size and percentage of fertile specimens are provided in Table 3.
2. The second experiment (EXP2‐Fl.Fr) evaluated the automated detection of flowers and fruits on herbarium specimens of angiosperms for our three test sets (A, B, C). Gymnosperms and ferns have been excluded from this experiment. This experiment extends one step beyond the previous one in terms of information precision, as it evaluated whether fertility is related to the presence of flower and/or fruit.
3. The third experiment (EXP3‐Pheno) dealt with the automated phenophase evaluation, which involved a higher number of visual classes. The test set of this experiment consisted of a random sampling of 20% of the original PHENO data set (the remaining 80% being used for training).

**Table 3:** Data distribution and results of the fertility detection accuracy obtained in EXP1‐Fertility.^a^

Evaluated models^b^	Training set	Test set A	Test set B	Test set C
Data set size	120,739	13,415	14,539	14,540
Percentage of fertile specimens	86.4%	86.2%	87.4%	91.1%
ResNet50‐Large (400 × 250 pixels)	—	94.9%	93.6%	93.2%
ResNet50‐VeryLarge (800 × 550 pixels)	—	96.3%	95.2%	92.0%

a Test set A = Random‐split, test set B = Species‐split (747 species), test set C = Herbarium‐split (nine NEVP herbaria).

b Default image size 900 × 600 pixels.

Data and models used and produced for this study are accessible on Zenodo (Lorieul, 2019; Lorieul et al., 2019), a free and open platform for preserving and sharing research output.

#### Comparing model results to secondary manual annotation

To compare results obtained by our four trained models (i.e., two models for the first experiment, one model for the second experiment, and a last model for the third experiment) to human expertise, the co‐author P.B., who had not previously been involved in annotating these data sets, manually annotated 100 herbarium specimens of each test set (a first subset of test set A used in EXP1‐Fertility and in EXP2‐Fl.Fr, a second subset from the test set used in EXP3‐Pheno). For the first (EXP1‐Fertility) and second (EXP2‐Fl.Fr) experiments, 100 herbarium specimens were randomly selected from test set A with a proportion of 25% of specimens from the four different categories: (a) true positives (i.e., with flower and/or fruit and correctly annotated by our model), (b) true negatives (i.e., without flower and/or fruit and correctly annotated by our model), (c) false positives (i.e., without flower and/or fruit and wrongly annotated by our model), and (d) false negatives (i.e., with flower and/or fruit and wrongly annotated by our model). The subset of specimens chosen for secondary manual annotation was potentially highly difficult to annotate by visual analysis of digitized specimens for a human, as it contains 50% wrongly annotated specimens by our model (categories c and d), and it was designed with such proportions in order to particularly inspect cases of automated annotation errors. For the third experiment (EXP3‐Pheno), 100 specimens randomly sampled from the test set were annotated by P.B., who did not use external resources to code phenophases.

## RESULTS

### Assessing performance of deep learning models

Results from the three experiments are provided in Tables 3, 4, 5, 6, and 7. Results of EXP1‐Fertility, presented in Table 3, show high performance for the correct detection of fertile material. Regardless of the strategy used to produce the three test sets, all achieved at least 92% correct detection. Performance of the models decreased from test set A (Random‐split) to test set B (Species‐split) and test set C (Herbarium‐split), but the performance gap between the three test sets is small, with, respectively, 1.7% and 4.3% difference between the best and worst performing ResNet50‐Large and ResNet50‐VeryLarge models. In addition, use of the ResNet50‐VeryLarge model slightly increased the number of correct detections for test sets A and B compared to ResNet50‐Large.

**Table 4:** Distribution of angiosperms, ferns, and gymnosperms in the data sets used for experiment EXP1‐Fertility and results of the fertility detection accuracy obtained in that experiment for test set A (Random‐split)

Evaluated clades	Data distribution					Fertility detection accuracy
	Whole data set	Training set	Test set A	Test set B	Test set C	Test set A
Angiosperms	91.47%	90.99%	90.49%	87.8%	100%	96.3%
Ferns and allies	8.51%	8.98%	9.47%	12.2%	0	95.7%
Gymnosperms	0.02%	0.03%	0.04%	0	0	100%

**Table 5:** Data distribution and results of the flower and fruit detection accuracy obtained in EXP2‐Fl.Fr

Evaluated models	Training set	Test set A (Random‐split)	Test set B (Species‐split)	Test set C (Herbarium‐split)
Data set size	109,467	12,095	12,723	14,066
Percentage of specimens in flower	60.9%	60.6%	62.5%	68.5%
Percentage of specimens in fruit	43.3%	43.4%	44.9%	32.5%
ResNet50‐flowers	—	84.3%	81.0%	87.0%
ResNet50‐fruits	—	80.5%	76.6%	79.6%

**Table 6:** Data distribution and results of the phenophase detection accuracy obtained in EXP3‐Pheno. Coarse classification accuracy is computed based on grouping phenophase categories by 3

Evaluated model	Training set size (No. of images)	Test set size (No. of images)	Fine‐grained classification accuracy	Coarse classification accuracy	Accuracy when tolerating a [±1] error range	Accuracy when tolerating a [±2] error range	Fine‐grained mean L1 error	Coarse mean L1 error
ResNet50‐Pheno	16,298	4073	43.4%	69.0%	67.1%	82.8%	1.35	0.37

**Table 7:** Data distribution and results of the phenophase detection accuracy obtained in EXP3‐Pheno, per phenophase categories

Phenophase	Data distribution in the training data set^a^	Data distribution in the test data set^a^	Classification accuracy
1	10.4%	10.4%	74.8%
2	7.6%	7.5%	24.4%
3	9.5%	9.5%	27.9%
4	7.1%	7.2%	8.6%
5	17.3%	17.2%	60.8%
6	7.2%	7.2%	6.8%
7	10.5%	10.6%	18.8%
8	10.5%	10.5%	18.0%
9	19.9%	19.9%	78.9%

a Human annotated.

**Figure 3:**
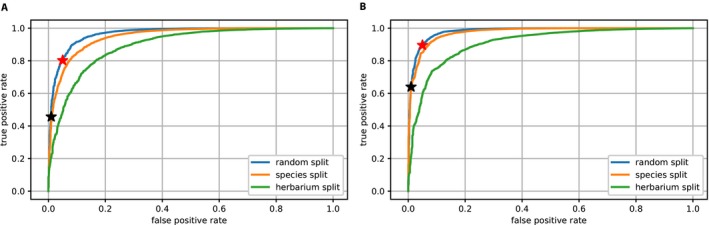
Fertility receiver operating characteristic (ROC) curves for EXP1‐Fertility, with ResNet50‐Large (A) and ResNet50‐VeryLarge (B). Blue = test set A (Random‐split); orange = test set B (Species‐split); green = test set C (Herbarium‐split); red stars = percentage of fertile specimens correctly detected at a false positive rate of 5%; black stars = percentage of fertile specimens correctly detected at a false positive rate of 1%.

The two models (i.e., ResNet50‐Large and ResNet50‐VeryLarge) showed distinct performances with each of the three test sets. Figure 3 shows the receiver operating characteristic (ROC) curves obtained for ResNet50‐Large (Fig. 3A) and ResNet50‐VeryLarge (Fig. 3B). At a false positive rate of 5%, the ResNet50‐Large model achieved a true positive rate of 80.3%, and the ResNet50‐VeryLarge model a true positive rate of 89.6%. At a false positive rate of 1%, the true positive rates of these models were 45.7% and 64.0%, respectively. These results highlight the importance of using higher‐resolution images for such tasks. Due to better performance of the ResNet50‐VeryLarge model, results provided in the remainder of this article (EXP2‐Fl.Fr and EXP3‐Pheno) are based on this model architecture.

**Figure 4:**
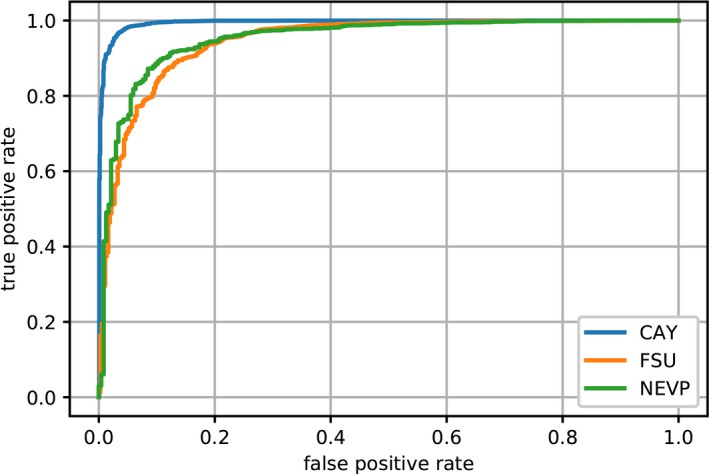
Fertility receiver operating characteristic (ROC) curves for EXP1‐Fertility, with ResNet50‐VeryLarge.

Results from test set A for angiosperms, gymnosperms, and ferns are provided in Table 4. Despite a low number of training images, the model achieved high performance with gymnosperms and ferns, with 100% and 95.7% correct detection, respectively. Figure 4 shows results from test set A for the NEVP, FSU, and CAY data sets. Detection of reproductive structures was more effective on specimens from the CAY data set than on specimens from NEVP and FSU. This can probably be explained by a combination of complementary factors such as: (1) a higher number of specimens per species in the CAY data set than the FSU data set (with a mean of 21 specimens per species in CAY and 14 specimens per species in FSU), and (2) highly visible reproductive structures of tropical and equatorial species compared to flowers and fruits of temperate species in the NEVP data set.

**Figure 5:**
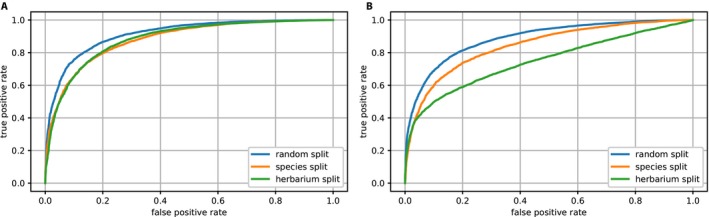
Flower detection (A) and fruit detection (B) receiver operating characteristic (ROC) curves for EXP2‐Fl.Fr, with ResNet50‐VeryLarge. Blue = test set A (Random‐split); orange = test set B (Species‐split); green = test set C (Herbarium‐split).

Regardless of the test set used, results of EXP2‐Fl.Fr (Table 5) show correct detection of the presence of flowers and fruits in more than 81.0% of cases for flowers and 76.6% for fruits. Flower detection was more efficient than fruit detection in the three test sets as shown in the ROC curves of Figure 5. It must be noted that there is a higher proportion of specimens with flowers than fruits in the training data as shown in Table 5. The model had more data to capture the concept of flower than fruit, resulting in lower fruit detection accuracy.

**Figure 6:**
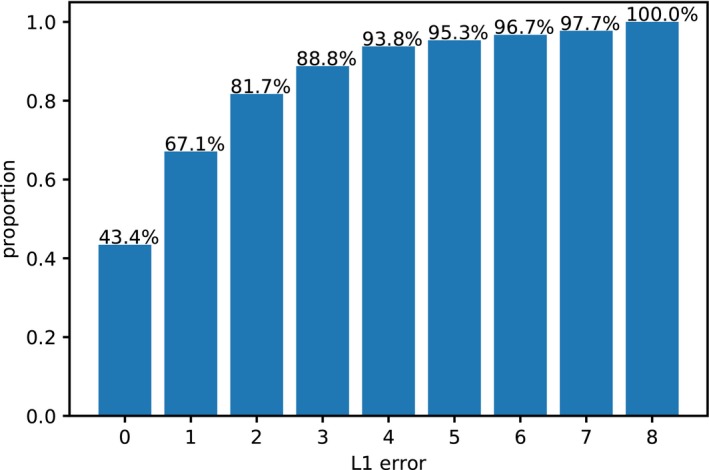
L1 error cumulative distribution for phenophase detection experiment (EXP3‐Pheno) using the PHENO data set.

**Figure 7:**
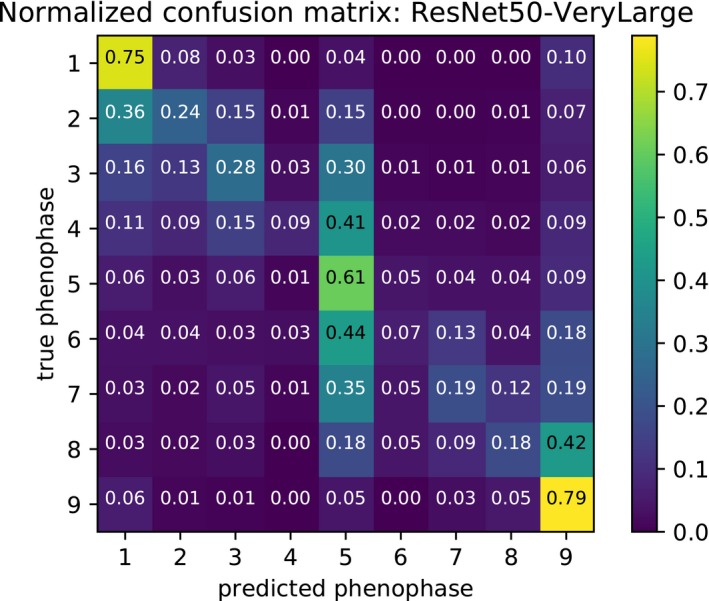
Row‐wise normalized confusion matrix of phenophase classification experiment (EXP3‐Pheno) using the PHENO data set.

Results from the fine‐grained phenology annotation (Table 6) conducted in EXP3‐Pheno show that correct fine‐grained classification accuracy is obtained in 43.4% of cases. This capacity increases to 69% for coarse classification accuracy. It is interesting to see that the best classification accuracy is provided for classes 1, 5, and 9, which correspond to all buds, peak flowering, and all fruit phenophases, respectively (Table 7). When we examine error distributions of these classifications (Fig. 6), we see that consistent classifications are obtained in 67.1% of cases with one class of error and in 81.7% of cases with two classes of error. The confusion matrix (Fig. 7) shows the most common confusions between phenophases. Phenophases 4 and 6 are the least well predicted. This is most likely related to the fact that they are the least common in the training data set, and they are visually very similar to phenophase 5. Indeed, these two phenophases have a potentially high percentage of flowering structures (more than 75%), whereas specimens of phenophase 5 have between 50% and 100% of their buds in flower. These three phenophases (i.e., phenophases 4, 5, 6) are also the only ones to combine (at different percentages) presence of buds, flowers, and fruits on the same specimens. All other categories involve specimens with a combination of two different reproductive attributes only (buds and flowers, buds and fruits, or flowers and fruits). For these reasons, most specimens of phenophases 4 and 6 could be easily mistaken as phenophase 5 by the model.

### Comparing model results to secondary manual annotation

The re‐annotation of 100 herbarium specimens of test set A from images of specimens resulted in 80% accuracy with original phenological annotations. We emphasize that this test subset is focused on some of the most difficult specimens to annotate, as 50% of them were wrongly annotated by our ResNet50‐VeryLarge model. The global accuracy of P.B. on the whole test set (87.8%) is computed using the average of the accuracy on each subset (categories a, b, c, d) weighted by their proportion in the whole test set. Accuracies for each category, overall accuracies on the test subset, and whole accuracies obtained by P.B. and the ResNet50‐VeryLarge model are provided in Table 8. Based on these results, we see that: (1) the reproduction of the original annotations was difficult for a human observer, and (2) the computation of the whole accuracy of the human observer (87%) shows a lower accuracy than that of the ResNet50‐VeryLarge model (96.3%).

**Table 8:** Comparison of model accuracy with human annotations based on the re‐annotation of 100 herbarium specimens of test set A of EXP1‐Fertility.^a^

Annotation types	True positive subset accuracy	False positive subset accuracy	True negative subset accuracy	False negative subset accuracy	Overall accuracy on these subsets	Global accuracy on test set A
ResNet50‐VeryLarge	100.0%	0.0%	100.0%	0.0%	50.0%	96.3%
Human annotation^b^	88.0%	68.0%	88.0%	76.0%	80.0%	87.8%

a The global accuracy on the whole test set is computed using the average of the accuracy on each subset weighted by their proportion in the whole test set, i.e., 84.1%, 1.7%, 12.2%, and 2.0%, respectively, for the true positive, false positive, true negative, and false negative subsets.

b Annotations were made by co‐author P.B.

The fact that the human observer (co‐author P.B.) did not achieve 100% accuracy on the subset of the 100 specimens of test set A can be explained by one of the following cases:

Case 1: Some specimens (6%) annotated as “fertile” in the original annotation were annotated as “non‐fertile” by P.B. because no reproductive structures were visible on the digitized specimen. This could be because (a) the original annotation was based on label text, which may indicate a state different from the specimen duplicate (e.g., the population or some of the specimen duplicates had reproductive material, but the particular duplicate examined did not), or (b) the flowers or fruits had disappeared during specimen manipulation/preparation (e.g., fallen off, been hidden from view in a fragment folder glued to the specimen, or been obscured by large leaves).
Case 2: Some specimens (6%) with closed reproductive buds were annotated “fertile” by P.B. but were annotated as “non‐fertile” by the original annotator.
Case 3: Some specimens (5%) described as “non‐fertile” by the original annotator were correctly detected as fertile by P.B.
Case 4: Some specimens (2%) were described as “fertile” on the label of the herbarium sheet and annotated as “fertile” by P.B., but they were annotated as “non‐fertile” by the original observer.
Case 5: One fertile specimen was not detected by P.B., due to the small size of the reproductive structures.

Cases 1 and 2 particularly highlight the fact that original annotations produced by the collectors, herbarium assistants, or digitization technicians can be different from annotations produced by the visual analysis of digitized specimens, even if all of them correctly followed a strict procedure. Case 1 is intrinsically related to herbarium management practices (that try to protect reproductive structures as much as possible, sometimes by hiding them in a folder), while case 2 is more related to definition and perception of the “fertile stage” on a plant specimen, which is sometimes hard to define, as reproductive structures are a continuum from tiny, closed buds to large, obvious fruits.

It is noteworthy that five of the six herbarium specimens annotated as “non‐fertile” by P.B. because no reproductive structures were visible (case 1) were similarly annotated by our trained model. Likewise, four of the five herbarium specimens annotated as “fertile” by P.B. in contrast to the incorrect original annotation (case 3) were annotated similarly by the trained model. This illustrates the potential of this technology to potentially detect incorrect annotations in herbarium databases. It should be noted that even if P.B. achieved 80% accuracy, the error rate in the original annotations of the very difficult subset of test set A is only 7% (cases 1, 2, and 5 cannot be considered as errors).

Human annotations of 100 randomly selected specimens from the PHENO test set by P.B., a non‐expert of that flora, offered complementary results. This secondary annotator achieved 42% and 68% accuracy with the original annotations with zero and one class of error, respectively. These results, very close to our trained model, highlight the difficulty of the task for a non‐expert who has not been trained on a particular taxonomic group, as well as the high likelihood for error when annotating specimens for fine‐scale phenological stages. More than 70% of inconsistent annotations from our model were also wrongly annotated by the secondary annotator. More than 25% of these errors were exactly the same and were mostly within one class of difference from the original annotation.

## DISCUSSION

These experiments clearly demonstrate the potential of deep learning technologies for automating phenological annotation of herbarium specimens. These promising results obtained for 7782 species of plants representing angiosperms, gymnosperms, and ferns suggest that it is possible to consider large‐scale phenological annotation across broad phylogenetic groups. Results obtained for the fertility detection experiment (EXP1‐Fertility, 96.3% consistent annotations) and for the flower and fruit experiments (EXP2‐Fl.Fr, 84.3% consistency for flower annotations and 80.9% for fruit annotations) are similar for the different test sets examined. This is encouraging, as it confirms that in the case of the results for test set B, trained CNNs are able to recognize visual features that illustrate fertility on plants, even if they are not learned on the same species in the training and testing data sets. As most herbarium data sets present a long tail distribution of their data (with most species represented by a small number of specimens), it is important to confirm high capacity of correct annotations even for rare species for which training data are often not available.

It is interesting and perhaps surprising that the models were more successful at detecting specimens with flowers present than specimens with fruit present. This result may be explained by several potential factors: (1) flowers can be more conspicuous because of their lighter colors, compared to dry fruits which are often darker than or a similar color to leaves or stems; (2) for a particular species, numbers of flowers on an inflorescence may be greater than the number of fruits, as several flowers can abort before developing into fruits; (3) mature fruits are often less well attached to the rest of the plant compared to flowers and can thus be easily lost during specimen preparation or handling; and (4) less data were available for the training phase of the fruit detection task than for the flower detection task.

Considering the difficulty of the task, the good performances achieved by models using data set C (Herbarium‐split) indicate that new data sets coming from herbaria that were not in the training data set can still be correctly annotated for phenology. Because managing conditions, specimen preparations, digitization parameters, and taxonomic annotations can vary considerably among different herbaria, this result is promising.

The similar results on the three different data sets (NEVP, FSU, and CAY) representing three distinct American floras demonstrate that our approach can be efficient for diverse plant groups from different environments and habitats and with highly distinct morphologies. The efficiency among the three studied clades (angiosperms, gymnosperms, and ferns) is also of great importance, demonstrating that this approach is effective on distant clades with highly dissimilar reproductive structures.

**Figure 8:**
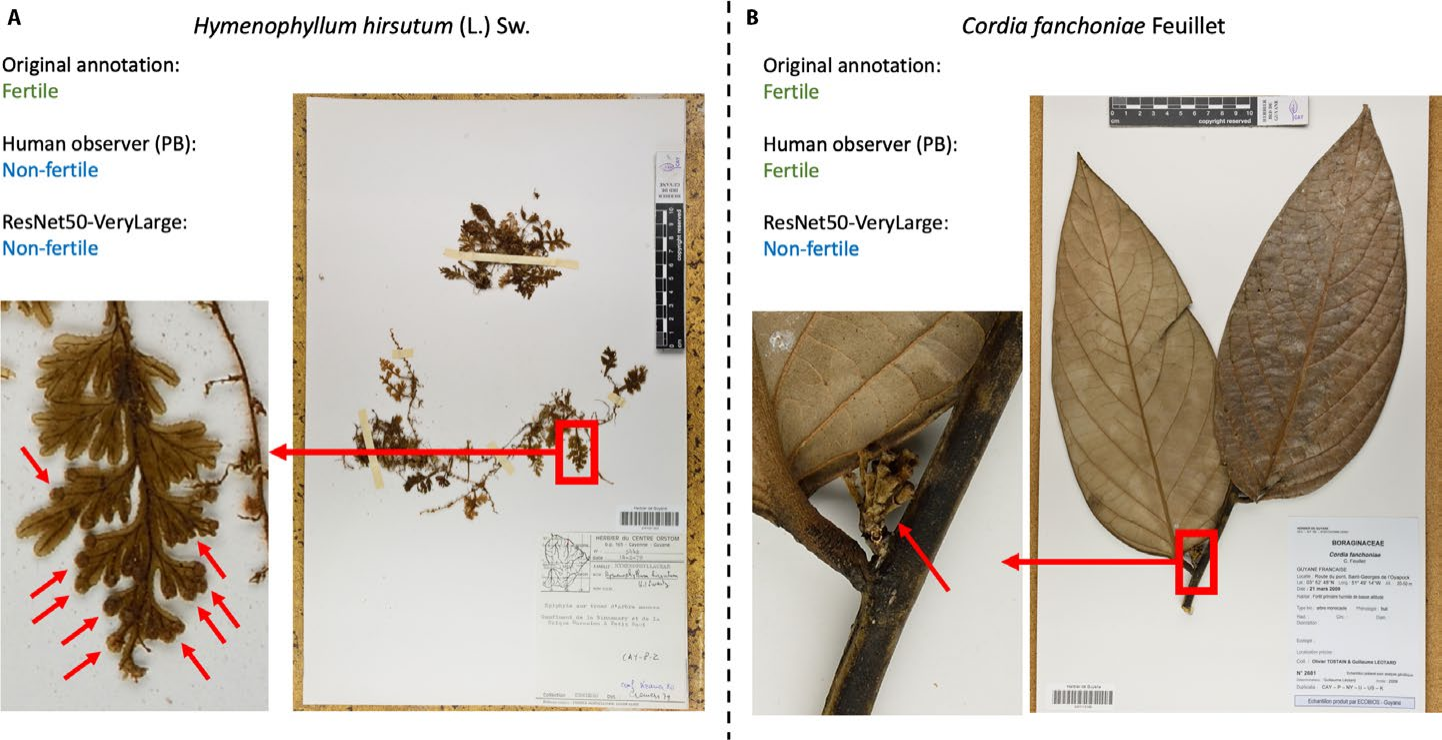
Illustration of some difficult specimens to annotate in EXP1‐Fertility. (A) Fertile specimen of *Hymenophyllum hirsutum*, wrongly annotated by the ResNet50‐VeryLarge model and the human observer (P.B.). Fertility is expressed by small terminal sori (1–1.5 mm in diameter) at the extremity of the lamina. Red arrows show them on a close‐up of the lamina. (B) Fertile specimen of *Cordia fanchoniae*, wrongly annotated by the ResNet50‐VeryLarge model and correctly annotated by the human observer (P.B.). Fertility is expressed by a small young infructescence (1.3 cm high, marked by red arrows), just after anthesis and before the development of fruits.

The work with complementary annotations provided by P.B. on a subset of test set A has highlighted the difficulty of the task for some of the most difficult specimens. Indeed, fertility is expressed by a wide variety of reproductive structures in terms of size, shape, color, etc. Furthermore, these reproductive structures are in continuous development and can be very inconspicuous at some development stages, as illustrated in Figure 8. Detection of fertility, in these cases, can be very difficult without the real specimen in hand. Because of the broad taxonomic coverage of the data analyzed, there are likely several taxa in the data set to which these circumstances apply.

The development of these technologies and their capacities to work on larger image sizes will undoubtedly benefit the herbarium, taxonomic, and research communities, as herbarium images are usually digitized and stored at very high resolution to permit investigation of small botanical characters. One strategy that could be explored to improve model performances is taking into account several different windows on each herbarium specimen, as CNNs are actually far from able to function at the original size of herbarium images. A complementary strategy could be to train models using complementary large, annotated data sets of observations of living plants in the field, which are now largely produced by networks of field botanists and/or amateur naturalists (e.g., iNaturalist or Pl@ntNet [Joly et al., 2016] networks), to enrich CNNs with specific images of fertile material. This work could open the door to new avenues of citizen science initiatives, such as annotation of phenological stages of living plants. Furthermore, as multimedia data streams are now much more easily produced by, e.g., drones equipped with cameras, such automated tasks could offer new opportunities for production of large volumes of phenological data.

The adoption of these automated techniques by collection managers, particularly within the framework of the established Plant Phenology Ontology (Brenskelle et al., 2019), could make it possible to (1) pre‐annotate large volumes of herbarium specimens that have not yet been annotated, which could then be revised by collaborative approaches; (2) have a standardized methodology that avoids bias related to expertise and perception variability of annotators; (3) use pre‐annotated herbarium specimens for phenological studies at large scales that would not be possible with human investment alone; and (4) identify and correct annotation mistakes made by human annotators.

The work on automated fine‐scale phenophase detection was carried out on species of Asteraceae, a group usually known for its small flowers and fruits, which cannot be easily detected on several specimens (as illustrated in Fig. 2). Such work on other plant families with more typical flowers and/or fruits (such as Fabaceae, Rosaceae, Rutaceae, and Bignoniaceae) could be informative as we can presume a stronger capacity of this approach to correctly detect fine phenological stages in these taxa. Other large herbaceous groups, such as Lamiaceae, Poaceae, or Cyperaceae, with completely different morphological attributes during their phenological cycles would also be interesting to evaluate using our model. The evaluation of the robustness of this fine‐grained phenological classification model on tree species could also be informative, as it could significantly improve the capacity to study forest phenological cycles. Pearson (2019b) and Ellwood et al. (2019) demonstrate that fine‐scale phenophases result in models that are more robust, yet without the use of a CNN annotating specimens for fine‐scale phenophases is a resource‐intensive task that is not likely to be broadly embraced. A possible way to improve performances of the model that we trained for phenophase detection, especially with an eye to research that is dependent on fine‐scale phenophases, could be to take into account the order of the phenophases and develop a potential counting system in the trained models. These are some of the strategies that will be investigated in our future work.

The work presented here opens the door for the adaptation of similar approaches to other detection and annotation tasks on botanical material such as (1) counting reproductive structures (e.g., flowers, inflorescences, leaves, fruits), especially in agricultural contexts for yield evaluation; (2) pathology detection (for plant pathologists interested in investigating disease on herbarium specimens, e.g., Meineke et al., 2018); and (3) annotation of morphological features of specimens, e.g., for rapid selection by professional taxonomists. New and enriched visual botanical data sets will undoubtedly contribute to progress on these challenges and will stimulate stronger exchanges between biological and computational sciences.

## Data Availability

Data and models used and produced for this study are accessible on Zenodo (Lorieul, 2019; Lorieul et al., 2019), a free and open platform for preserving and sharing research output.

## Details of the herbarium specimen annotation process.

Data collected on herbarium specimens in this study were obtained according to the following method:

1. Plant specimens were collected in the field by a collector, who recorded information on the collecting location, date, and potential scientific name of the specimen. Additionally, a collector may have recorded a description of the environment in which the plant was growing and possibly a description of the plant specimen itself, including details about the plant's reproductive condition. In common botanical practice, a plant specimen is considered a gathering, or part of a gathering, of a single species or infraspecific taxon made at one time by a particular collector at a particular place. Thus, a specimen may consist of multiple individuals of the same taxon (each of these samples is called a “specimen duplicate”). In such cases, it is important to remember that the data provided by the collector are not necessarily a description of an individual specimen duplicate. This can result in a difference between the description on the specimen label and the visible information on the specimen duplicate in the herbarium.
2. Collector sends his specimens to one or several herbaria, accompanied by a label with the collector‐provided data mentioned above.
3. Physical specimens are mounted on herbarium sheets with a herbarium label. Information from the herbarium label is then transcribed by herbarium personnel into an electronic database. In some workflows, details about the reproductive condition reported on the label are recorded (e.g., into a specific field or into a field containing the entire plant description).
4. The specimen‐level information recorded in the herbarium database system can be updated when the herbarium specimen or image of the specimen is studied after its initial preparation. These updates can include annotations related to identification (based on opinions of experts who have analyzed the specimen). Annotations related to the reproductive condition of the specimens can also be performed if, for example, that information was not provided on the label or was not recorded in the initial database transcription step. In some rare cases, an annotation may report that a herbarium specimen is without reproductive structures, while the label notes that plant was observed in the field with reproductive attributes. This second example can be understood by several possibilities: (1) the collector was not able to collect a fertile part of the plant (e.g., the fertile material was too high or too far from the collector, which can be often the case in tropical forests), (2) not all of the duplicates have fertile material, or (3) flowers and fruits can be fragile and become separated from the specimen when specimens are old or frequently manipulated.
5. Our data are based on the most recent annotations present in institutional herbarium data management systems or in collaborative data sharing portals (e.g., in the case of NEVP). These annotations are not necessarily produced by the collector, but by various trained herbarium personnel.

## List of institutions contributing data to the NEVP phenology data set. Institution abbreviations are according to Index Herbariorum (Thiers, 2016).

**Table:**

Institution names^a^	Institution codes
Arnold Arboretum, Harvard University	A
Bartlett Arboretum	BART
Boston University	BSN
Brown University	BRU
Connecticut Botanical Society	NCBS
Central Connecticut State University	CCSU
Connecticut College	CCNL
University of Connecticut	CONN
Economic Herbarium of Oakes Ames, Harvard University	ECON
Farlow Herbarium, Harvard University	FH
Gray Herbarium, Harvard University	GH
Keene State College	KESC
New York Botanical Garden	NY
New England Botanical Club	NEBC
Rutgers University	CHRB
University of Maine	MAINE
University of Massachusetts	MASS
University of New Hampshire	NHA
University of Rhode Island	KIRI
University of Vermont	VT
Western Connecticut State University	WCSU
Westfield State University	WSCH
Wilton Garden Club	WGCH
Yale Peabody Museum of Natural History, Yale University	YU

a All institutions are located in the United States.

## List of institutions contributing data to the PHENO data set. Institution abbreviations are according to Index Herbariorum (Thiers, 2016).

**Table:**

Institution names^a^	Institution codes
Archbold Biological Station	ARCH
Arizona State University	ASU
Ohio University	BHO
Centre County Historical Society	CCHS
The College of Idaho	CIC
Central Michigan University	CMC
University of Connecticut	CONN
Converse College	CONV
Denver Botanic Gardens	DBG
Desert Botanical Garden	DES
Eastern Illinois University	EIU
The Ronald L. Jones Herbarium	EKY
Eastern Michigan University	EMC
Florida Museum of Natural History	FLAS
Florida State University	FSU
University of Georgia	GA
Gray Herbarium, Harvard University	GH
Georgia Southwestern State University	GSW
Hope College	HCHM
Portland State University	HPSU
University of Idaho	ID
Idaho State University	IDS
University of Illinois	ILL
Illinois Natural History Survey, Illinois Dept. of Natural Resources	ILLS
Keene State College	KESC
University of Rhode Island	KIRI
Louisiana State University	LSU
University of Michigan	MICH
University of Minnesota	MIN
University of Mississippi	MISS
Mississippi State University	MISSA
Missouri Botanical Garden	MO
Morton Arboretum, Illinois	MOR
Marshall University	MUHW
North Carolina State University	NCSC
University of North Carolina at Chapel Hill	NCU
Natural History Museum of United Kingdom	NHMUK
New York Botanical Garden	NY
Marie Selby Botanical Gardens	SEL
Boise State University	SRP
Troy University	TROY
Tall Timbers Research Station	TTRS
James F. Matthews Center for Biodiversity Studies	UNCC
University of New Mexico	UNM
Smithsonian Institution	US
University of South Carolina	USCH
University of South Carolina Upstate	USCS
University of South Florida	USF
University of Southern Mississippi	USMS
Intermountain Herbarium, Utah State University	USU
University of West Florida	UWFP
University of Wisconsin–La Crosse	UWL
University of Wisconsin–Milwaukee	UWM
Valdosta State University	VSC
Western Carolina University	WCUH
Whitman College	WCW
University of Wisconsin–Madison	WIS

a All institutions are located in the United States.

## Details of the ResNet50 adaptation to larger image size.

Like most image classification architectures, the original ResNet50 model consists mainly of a succession of convolution layers followed by a spatial pooling and a final fully connected layer. The convolution layers act as visual feature extractors, the pooling layer removes the residual spatial information, and the fully connected layer performs the final classification in the new feature space. Unlike this final layer, the convolution layers are agnostic to the spatial extent of their inputs. However, in transfer learning, the final fully connected layer is learned from scratch because it is specific to each task. Thus, to adapt the ResNet50 architecture to larger image size, we modified the spatial pooling layer in order to perform this pooling operation in the total spatial extent, which has increased due to the new image size. By doing so, we kept the input size of the fully connected layer the same as the original model while being able to exploit finer details in the images. Such a modification can be applied to many other architectures and is not limited to ResNet50.

## Details of the fine‐tuning procedure.

Instead of initializing the parameters with random values, we used the pre‐trained weights available from PyTorch's model *zoo*. We then fine‐tuned this model on our data sets by optimizing the binary cross‐entropy for EXP1‐Fertility and EXP2‐Fl.Fr and the multinomial cross‐entropy for EXP3‐Pheno. As optimizer, we used stochastic gradient descent (SGD) with momentum. We kept 10% of the training data to build a validation set that was then used to choose the values of the hyperparameters of SGD. We detail these values in the next paragraph.

For every experiment, we used a fixed learning rate decay: the initial learning rate value was divided by 10 at one‐third and two‐thirds of the training. Nesterov's momentum was used with a value of 0.9. No weight decay was performed. The values of the initial learning rate, the batch size, and the number of epochs were different depending on the experiment. Details of these values are provided in Table A5‐1.

The gap in batch size between ResNet50‐Large and ResNet50‐VeryLarge was mainly due to memory restrictions of the graphics processing units (GPUs). Indeed, although using higher‐resolution images does not impact the number of parameters in the model, it did result in more activations being computed during training: four times more memory was needed for ResNet50‐VeryLarge than for ResNet50‐Large. The difference in number of epochs was explained by the fact that EXP3‐Pheno has fewer images than EXP1‐Fertility and EXP2‐Fl.Fr.

Before being fed to the model, for ResNet50‐Large, the images were resized to 450 × 300 pixels (approximately, because their ratio was preserved), and they were cropped according to a centered rectangle of 400 × 250 pixels. Similarly, for ResNet50‐VeryLarge, they were approximately resized to 900 × 600 pixels and cropped down to 850 × 550 pixels. Moreover, data augmentation was used by performing random horizontal and vertical flips and random rotations of ±45 degrees on the input images during training.

**Table A5‐1:** Values of the initial learning rate, the batch size, and the number of epochs for each experiment

Experiment	Initial learning rate	Batch size	No. of epochs
EXP1‐Fertility ResNet50‐Large	0.001	48	45
EXP1‐Fertility ResNet50‐VeryLarge	0.001	12	45
EXP2‐Fl.Fr ResNet50‐VeryLarge	0.01	12	45
EXP3‐Pheno ResNet50‐VeryLarge	0.001	8	30
